# The evolving role of GLP-1 agonists in ischemic stroke prevention in diabetic patients

**DOI:** 10.1097/XCE.0000000000000308

**Published:** 2024-08-14

**Authors:** Aditi Shankar, Aditi Sharma, Chirag Buch, Robert J. Chilton

**Affiliations:** 1 Division of Cardiology, Department of Medicine, University of Texas Health San Antonio, San Antonio, Texas, USA

The incidence of strokes continues to rise steadily, reaching almost 800 000 cases annually, with 87% of them attributed to ischemic causes [1]. A recent analysis of a post hoc prospective trial involving 701 participants from the Prevention of Cardiovascular Events in Ischemic Stroke Patients with High Risk of Cerebral Hemorrhage (PICASSO) trial focused on individuals with newly diagnosed diabetes. The study revealed that the rate of ischemic stroke per 100 person-years was 8.93 for those with newly diagnosed diabetes, 3.79 for those with known diabetes, and 2.64 for those without diabetes. These differences were statistically significant (*P* = 0.0092) [2].

In summary, the increasing prevalence of individuals consuming excessive calories is leading to a rise in cardiometabolic diseases. This, in turn, contributes to a notable increase in the occurrence of ischemic strokes, particularly in individuals recently diagnosed with diabetes.

The primary focus of stroke research has shifted toward the penumbra region in recent years. ‘Time is brain’ concept for the brain health principle underscores the significance of the ischemic penumbra in improving stroke outcomes. Addressing multiple risk factors associated with cardiometabolic diseases has emerged as a crucial strategy for preventing stroke and vascular events in diabetes patients. Notably, the brain, which depends heavily on glucose for energy and constitutes 25% of basal metabolism is highly sensitive to a loss of blood supply.

Diabetes increases the risk of stroke through various postulated mechanisms, including vascular endothelial dysfunction, systemic inflammation, increased arterial stiffness at an early age, and increasing the thickness of vascular beds [3].

Brain tissue reacts swiftly to an ischemic event, with brain cells beginning to die within 5 min, compared to cardiac cells which take about 20–40 min. Furthermore, the brain is more prone to neuron damage due to intracellular signaling pathways that amplify energy failure and oxidative stress. The acute deprivation of oxygen and glucose leads to two detrimental changes in neurons: the release of the excitatory transmitter glutamate, causing cell necrosis, and a surge in calcium influx, leading to apoptosis. Both excitotoxic necrosis and apoptosis occur simultaneously [4–6]. Glucagon-like peptide-1 receptor (GLP-1R) agonists have been approved for the management of diabetes due to their effect on glucose homeostasis and satiety regulation. The GLP 1R agonists exert their action by enhancing glucose-mediated insulin release from beta cells of the pancreas [7], decreasing glucose production potentially by its action on alpha cells [7] Also, by regulating food intake and energy expenditure by the effect on brown adipose tissue metabolism [8] and by delaying gastric emptying [9].

Numerous large-scale clinical trials suggest that GLP-1R agonists have the potential to reduce stroke incidence, although the precise mechanism remains under intensive investigation. One hypothesis is that GLP-1R agonists may modify the excitotoxic effects of glutamate. However, basic research indicates that GLP-1R agonists can increase glutamate release [10,11]. Other critical neuroprotective mechanisms associated with GLP-1R agonists require further exploration. The central nervous system plays a pivotal role in cardiovascular and neurological protection, involving elements such as the parasympathetic system, venous blood flow, and nitric oxide [8]. At the cellular level, stroke-induced changes are comprehensive. During brain ischemia, endothelial cells release vascular endothelial growth factor A, which exacerbates blood–brain barrier leakage. Astrocytes contribute to this by releasing matrix metalloproteinases 9, interleukin (IL)-1, IL-6, and tumor necrosis factor alpha, further compromising the blood-brain barrier (Fig. 1). Fortunately, basic studies indicate that GLP-1 agonists can mitigate the toxic secretions from astrocytes [15].

**Fig. 1:**
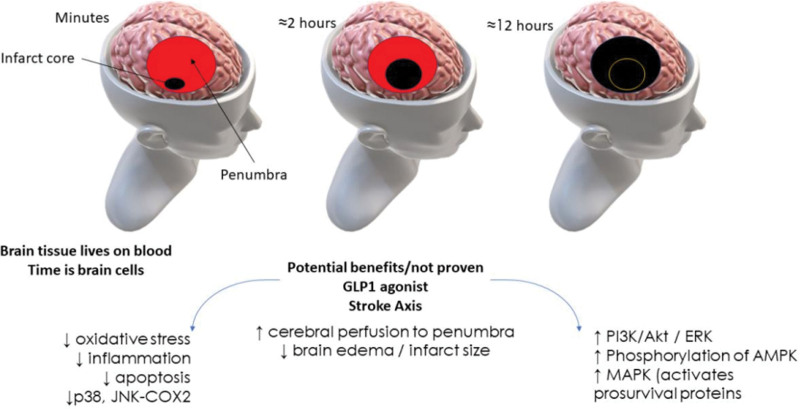
Illustrates the potential ways in which GLP-1-R agonists could offer stroke risk reduction benefits, based on recent trials. Regrettably, there is a scarcity of data supporting clear mechanisms. Additionally, astrocytes’ release of inflammatory elements such as MMPs, chemokines, and cytokines during stroke leads to inflammation, but these effects can be mitigated by GLP-1R agonists, as indicated by basic research. Adapted from [12–14]. GLP-1R, glucagon-like peptide-1 receptor; MMPs, matrix metalloproteinases.

A comprehensive review of published evidence regarding GLP-1R agonists in stroke reduction highlights several randomized control trials (Lixisnatide in Patients with Type 2 Diabetes and Acute Coronary Syndrome, Liraglutide and Cardiovascular Outcomes in Type 2 diabetes [LEADER], Semaglutide and Cardiovascular Outcomes in Patients with Type 2 Diabetes [SUSTAIN], Effects of Once Weekly Exenatide on Cardiovascular Outcomes in Type 2 Diabetes, and Albiglutide and Cardiovascular Outcomes in Patients with Type 2 diabetes and Cardiovascular Disease) and multiple meta-analyses. Notably, a meta-analysis by Malhotra *et al*. [16] demonstrated that GLP-1R agonist treatment significantly reduced the odds of nonfatal strokes in patients with type 2 diabetes. A pooled analysis by Barkas *et al*. [17] found that GLP-1R agonists reduced the total risk of stroke by 13%. Furthermore, specific trials such as SUSTAIN-6 and LEADER showed substantial reductions in stroke risk with GLP-1R agonist (GLP-1RA) treatment [17,18]. Moreover, the LEADER trial (Liraglutide and Cardiovascular Outcomes in Type 2 Diabetes) by Marso *et al*. [19], showed that the primary composite outcome occurred in fewer patients in the liraglutide group [608 of 4668 patients (13.0%)] than in the placebo group [694 of 4672 (14.9%)] (*P* < 0.001) [19]^.^

In a more recent meta-analysis by Wei *et al*. [20] ischemic stroke events were significantly reduced with GLP-1RA versus control (*P* < 0.008; 95% CI, 0.73–0.95). However, hemorrhagic strokes were not significantly different (*P* = 0.31) (Fig. 2).

**Fig. 2:**
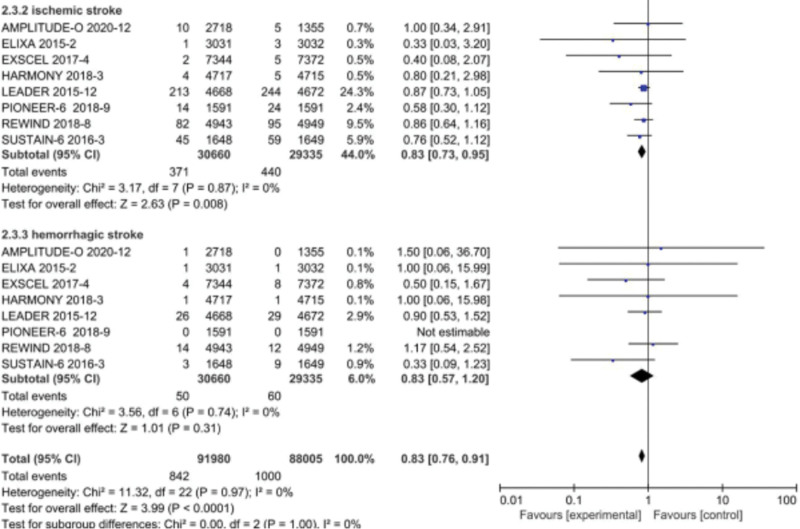
GLP-1 RA finds benefit in reducing ischemic stroke but no increase or benefit in hemorrhagic strokes. Adapted from [17].

In conclusion, individuals with diabetes face a heightened risk of ischemic stroke, and treatment with GLP-1R agonists may potentially provide stroke-related benefits. However, while some important clinical trials have demonstrated clear benefits, this area of research remains open and necessitates further investigation.

## Conflicts of interest

There are no conflicts of interest.
